# Primary and Secondary Genotoxicity of Nanoparticles: Establishing a Co-Culture Protocol for Assessing Micronucleus Using Flow Cytometry

**DOI:** 10.3389/ftox.2022.845987

**Published:** 2022-03-08

**Authors:** N. V. Srikanth Vallabani, Hanna L. Karlsson

**Affiliations:** Institute of Environmental Medicine, Karolinska Institute, Stockholm, Sweden

**Keywords:** NiO nanoparticles, HBEC3-kt cells, THP-1 cells, macrophages, cell cycle analysis, DNA damage, micronuclei, metal oxide

## Abstract

Genotoxicity is an important endpoint to assess for understanding the risks associated with nanoparticles (NPs). Most genotoxicity studies performed on NPs have focused on primary genotoxicity analyzed by comet- or micronuclei (MN) assay using microscopic scoring. Here, we established a protocol for a more efficient version of MN assessment using flow cytometry and, importantly, both primary and secondary (inflammation-driven) genotoxicity was assessed. Human bronchial epithelial cells (HBEC-3kt) were exposed to nickel oxide (NiO) NPs directly or indirectly. The indirect exposure was done to assess secondary genotoxicity, and in this case immune cells (THP-1 derived macrophages) were exposed on inserts and the HBEC were cultured in the lower compartment. The results in monocultures showed that no increased MN formation was observed in the HBEC cells but instead a clear MN induction was noted in THP-1 cells indicating higher sensitivity. No MN formation was either observed when the HBEC were indirectly exposed, but an increase in DNA strand breaks was detected using the comet assay. Taken together, the present study emphasizes the feasibility of assessing primary and secondary genotoxicity and, furthermore, shows a clear MN induction in THP-1 monoculture following NiO NPs exposure.

**GRAPHICAL ABSTRACT:**
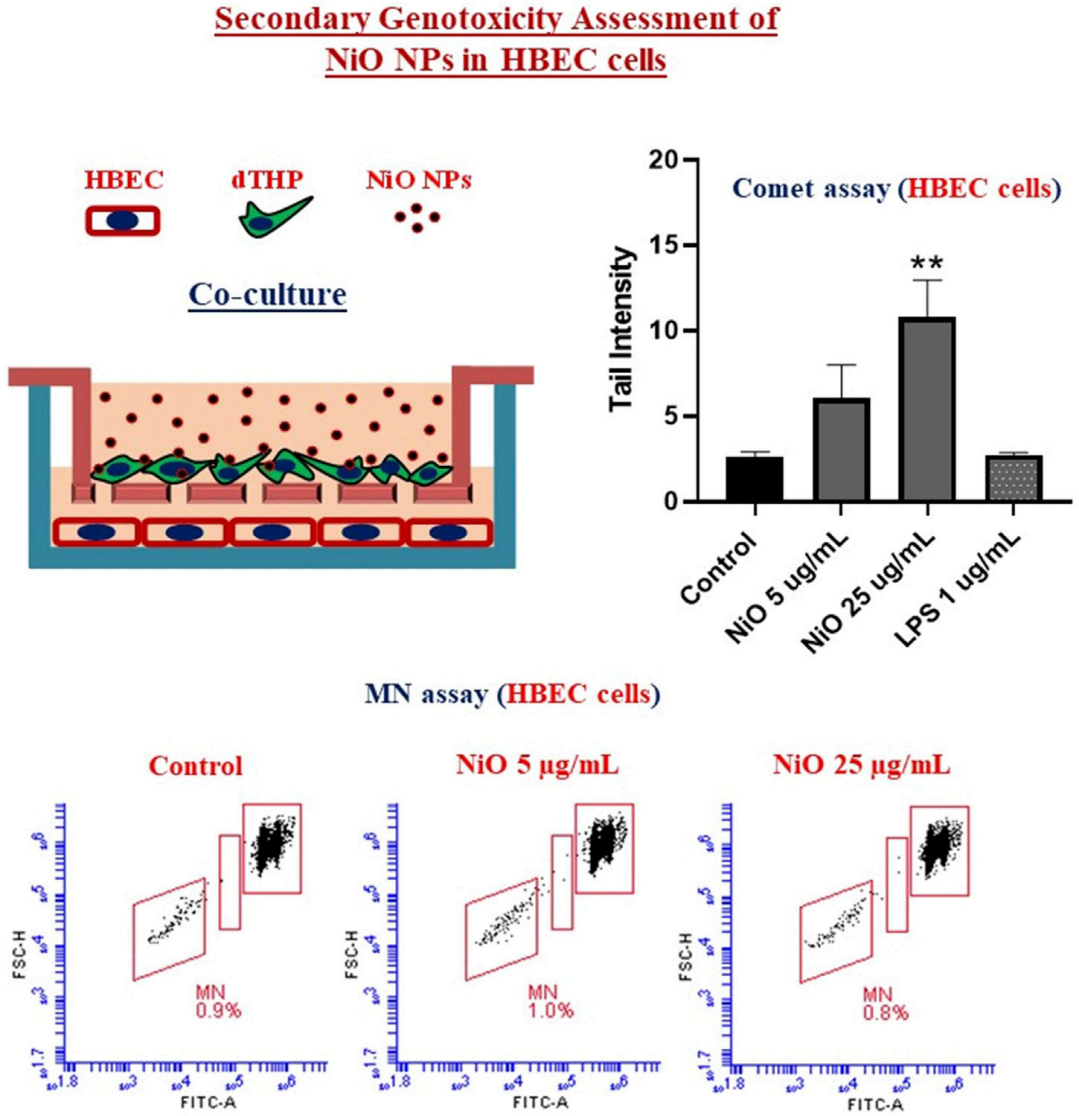


## Introduction

Nanoparticles (NPs) possess distinct physicochemical properties, and their unique characteristics makes them novel entities for a wide range of applications in medicine, engineering, pharmaceuticals, cosmetics, and electronics etc., (Salata, 2004; Ealia and Saravanakumar, 2017). However, their extensive production and usage lead to a demand for toxicity evaluation as well as assessment of health risks at occupational and environmental settings (Kessler, 2011; Batley et al., 2013). Among the various NPs, nickel oxide (NiO) is vastly employed in industrial applications such as metal printing, electronics, ceramics, catalysis, and sensing (Zhou et al., 2017; Sousa et al., 2018; Dumala et al., 2019; Jaji et al., 2020; Taeño et al., 2021; Wang et al., 2021). Moreover, exposure is common at nickel refineries, metal alloy production sites and at occupational setting where welding is performed (Klein and Costa, 2015; Pesch et al., 2019). The health risks possibly caused by inhalation of nickel is evident since nickel compounds are classified as carcinogenic to humans (Group 1) by the International Agency for Research on Cancer. Furthermore, several studies have shown genotoxicity following exposure of lung cells to NiO NPs (Capasso et al., 2014; Di Bucchianico et al., 2018; Akerlund et al., 2019).

Genotoxicity testing typically includes a battery of assays and in a first tier testing various *in vitro* assays are performed (Dusinska et al., 2019). The most commonly used assays in genotoxicity testing of NPs are Micronucleus (MN) assay and Comet assay, respectively (Magdolenova et al., 2014). They are often used in combination due to their advantages over each other, where comet assay detects the DNA damage/stand breaks with high sensitivity, and MN assay can differentiate the aneugenic and clastogenic effects (Magdolenova et al., 2014; Franz et al., 2020). In the conventional MN method microscopic scoring is mainly used to score at least 2000 binucleate cells per concentration. This evaluation is tedious and a time-consuming process. Hence, high throughput methods like flow cytometric MN analysis can be employed to automate the analysis. Furthermore, the sequential staining procedure allows discriminating the actual MN compared to MN originated from dead or dying cells (necrotic/apoptotic population) (Avlasevich et al., 2006; Bryce et al., 2007).

One aspect often not considered in standard genotoxicity assessment is the so-called secondary genotoxicity. In general, secondary genotoxicity is exhibited *in vivo* via inflammation mediated mechanisms caused by activation/recruitment of phagocytes (macrophages or neutrophils). For instance, the presence of foreign bodies or uncleared NPs in lung cells can elicit a chronic immune response involving oxidative stress (ROS and reactive nitrogen species). The whole cascade triggers free radical generation, and cytokine/chemokine release from immune cells causing secondary genotoxicity towards neighboring target cells. Some advanced *in vitro* approaches have been used to mimic the *in vivo* conditions and understand the secondary genotoxicity mechanisms (Evans et al., 2017; Akerlund et al., 2019; Evans et al., 2019; Burgum et al., 2021). These approaches include application of conditioned culture medium from one cell type to other target cells, co-culture systems to facilitate cell-to-cell interplay among different cell types, and complex 3D cellular microtissues (spheroids or organoids) models which resemble *in vivo* tissue architecture and characteristics. However, only a limited number of studies have attempted to investigate secondary genotoxicity (Evans et al., 2017; Akerlund et al., 2019; Evans et al., 2019). Similarly, only few studies have used flow cytometry for more efficient analysis of MN induction following exposure to nanoparticles (Di Bucchianico et al., 2017; Lebedova et al., 2018). The aim of this study was to establish a flow cytometry protocol for MN analysis that is useful for detecting primary and secondary genotoxicity of NPs. A co-culture model with macrophages and lung cells was used to determine the secondary genotoxicity of NiO NPs. The MN formation using the flow cytometer approach was also compared to the results achieved with the comet assay for assessment of DNA strand breaks. Importantly, possible interferences were also considered.

## Materials and Methods

### Cell Culture

HBEC3-kt (Human bronchial epithelial cells) were originally obtained from American Type Culture Collection (ATCC) and were cultured in 50% LHC-9 (Laboratory of Human Carcinogenesis-9, Gibco, Carlsbad, CA) and 50% RPMI medium (Roswell Park Memorial Institute, Sigma Aldrich, St. Louis, MO) without serum and supplemented with 1% penicillin-streptomycin (Gibco, Buffalo, NY) and 2 mM L-glutamine (Gibco, Buffalo, NY). Prior to cell culture T75 flasks were coated with 3 mL of collagen (0.032 mg/mL, Type I, PureCol^®^, Advanced BioMatrix Carlsbad, CA) for 2 h, and cells were maintained at 37°C in a humidified incubator supplied with 5% CO_2_.

THP-1 monocytes (THP) were obtained from Sigma-Aldrich and cultured in RPMI-1640 medium supplemented with 10% FBS, 2 mM L-glutamine and 1% penicillin-streptomycin. Cells were grown in a T75 cm^2^ flask (VWR 734-2313) and incubated at 37°C in a humidified incubator supplied with 5% CO_2_. Cell density was maintained between 5 × 10^5^–1.5 × 10^6^ cells/mL. THP were differentiated to macrophages (dTHP) with 50 ng/mL phorbol 12-myristate 13-acetate (PMA, Sigma) at 37°C for 48 h.

### Particle Preparation and Characterization

NiO NPs (<50 nm diameter, >99.8% purity, Cat# 637130, 17198PJ) were purchased from Sigma-Aldrich (St. Louis, MO). NPs were weighed and dispersed in Milli-Q water to make a stock concentration of 1 mg/mL. The suspension was then sonicated in a water bath sonicator (VWR, USC 200T) for 20 min at 30°C, and then further diluted in cell medium to the indicated concentrations. Detailed characterization of the NiO NPs has been presented in our earlier publications (Di Bucchianico et al., 2018; Akerlund et al., 2019).

### Alamar Blue Assay

THP-1 cells were seeded at a density of 2.0 × 10^4^ cells/well in a 96 well plate. HBEC cells (1.0 × 10^4^/well) were seeded in a collagen precoated 96 well plate and incubated for 24 h. Both cell types were exposed to NiO NPs at 5, 10, and 25 μg/mL for 48 h in their respective medium and cell culture medium was used as a negative control. After exposure, supernatant was removed from HBEC and 10% Alamar Blue (Invitrogen, Carlsbad, CA) prepared in fresh medium was added. In case of THP-1 cells, Alamar blue was added directly into the existing medium (to make a final concentration of 10%) and incubated for 2 h at 37°C. Wells containing only 10% Alamar blue and NiO NPs were included to rule out particle interference in the study. After incubation, the fluorescence was read at 560 nm excitation and 590 nm emission using a microplate reader (Tecan, San Jose CA, Infinite F 200, Software: Magellan 7.2). Negative control was normalized to 100% viability and treated samples were compared with this value.

### Co-Culture Using Inserts

The differentiation of THP-1 to macrophages (dTHP) was carried by incubating 5.0 × 10^5^ cells/insert in 400 µL of medium containing PMA. Inserts [ThinCert™ PET membrane inserts (Greiner Bio-One, 662641), pore size 0.4 µm, surface 0.33 cm^2^] with cells were placed in a 24 well plate and allowed to differentiate for 48 h. HBEC cells at a density of 0.6 × 10^5^ cells (600 µL/well) were seeded in 24 well plate and left for 24 h, fresh HBEC medium was replaced before placing the inserts. Prior to exposure, dTHP were washed gently with PBS and inserts were placed on top of HBEC cells. The dTHP were then exposed to NiO NPs for 24 or 48 h in RPMI medium, unexposed cells were considered as negative control.

After exposure, inserts were removed and dTHP cells were preceded for cytotoxicity testing. Alamar blue was added to the medium, after 2 h supernatant from inserts was transferred to a 96 well plate and fluorescence intensity was recorded. HBEC cells were assessed for secondary genotoxicity using micronucleus and comet assays.

### Micronucleus Assay

Micronucleus detection was followed by a flow cytometric method described by Bryce et al., with some modifications (Bryce et al., 2007).

#### Monocultures and Primary Genotoxicity

HBEC cells (0.6 × 10^5^ cells) were exposed to NiO NPs (5, 10, and 25 μg/mL) for 48 h in a 24 well plate. After treatment cells were washed with chilled PBS and continued to step 1–3 as described below. In case of suspension cultures (THP-1) 1.2 × 10^5^ cells were seeded in a 24 well plate, and after NPs incubation cells were centrifuged at 1,500 rpm for 5 min. Supernatant was discarded, cells were washed with PBS and centrifuged again to collect the pellet. Further, cells were processed to step 1–3 before analysis.

#### Coculture and Secondary Genotoxicity

After exposure to NiO NPs (5 and 25 μg/mL) the inserts containing dTHP were removed and, HBEC cells from co-culture were transferred on to ice and left for 20 min. Next the medium was removed, and cells were washed with ice-cold PBS.

Step 1: Ethidium Monoazide Bromide dye (EMA, Invitrogen) stock was prepared in DMSO, and the working concentration (10 μg/mL) was prepared in buffer solution (PBS+2% FBS). EMA solution (300 µL) was added to cells and incubated on ice for 30 min, under a cool white light. After incubation cells were washed with ice-cold buffer solution and continued for step 2.

Note: For suspension culture (THP-1), after EMA staining cells were centrifuged at 1,500 rpm for 5 min and pellet was dispersed in buffer solution to wash. Thereafter, cells were centrifuged to collect the cell pellet and processed for step 2.

Step 2: Lysis solution I (Trisodium citrate 1.0 mg/mL; NaCl 0.584 mg/mL; Igepal 0.6 μL/mL; RNase A 100 μg/mL and SYTOX Green 0.5 μM) was prepared in Milli Q and filtered using 0.22 µm pore size membrane filter. To each well 300 µL of lysis buffer (solution I) was added and incubated in dark for 1 h at room temperature.

Step 3: Solution II (Citric acid 15 mg/mL; sucrose 85.6 mg/mL and SYTOX Green 0.5 μM) was prepared in Milli Q and filtered using 0.22 µm pore size membrane filter. 300 µL of solution II was added to the cells (without discarding solution I) and was allowed to equilibrate in dark for 30 min at room temperature.

Additional step: A drop of cell sorting set-up beads (6 μm, for blue lasers, Invitrogen) can be mixed in solution II (∼10 mL) prior adding to cells. Based on the healthy nuclei to bead ratio, cytotoxicity can be calculated from the relative survival values.

#### Advantage of Differential Staining

**SCHEME 1:**
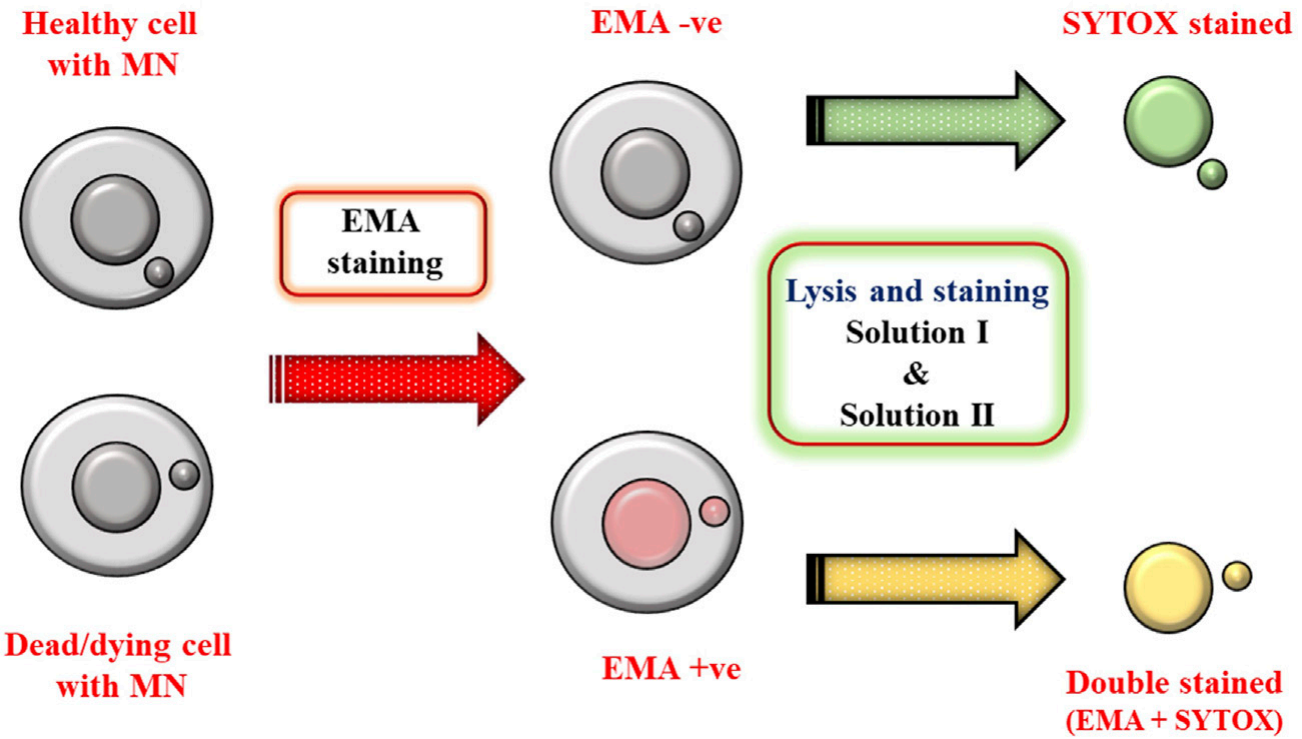
Schematic describing the advantage of differential staining in excluding dead/dying cells (EMA +ve population).

EMA dye enters the cells which have compromised membrane and binds covalently to nucleic acids after photolysis. Up on binding to nucleic acids, its fluorescent intensity increases and can differentiate live and dead cells in a mixed population. After EMA staining, the detergent in solution lyses the cytoplasmic membrane of the cells and liberates nuclei and MN. Concomitantly, SYTOX Green stains the overall DNA and this differential staining procedure helps to rule out the dead/dying cells (double positive) compared to healthy cells. Based on staining, healthy cells are termed as EMA-negative (EMA −ve) and dead cells as EMA-positive (EMA +ve) population. For Flow cytometric analysis, only EMA −ve nuclei were considered for MN evaluation to exclude necrotic or apoptotic population (see Scheme 1).

**SCHEME 2:**
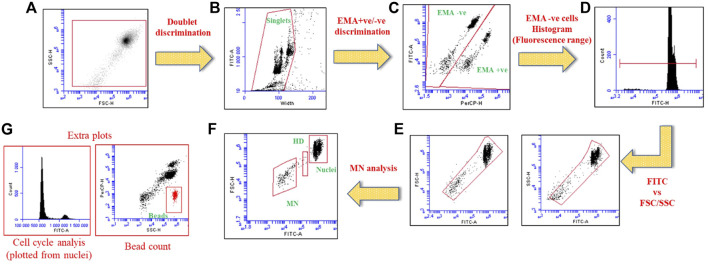
Dot plots and histograms illustrating the analysis setup for MN detection using flow cytometric method. **(A)** Total gated population, **(B)** Double discrimination to choose singlet nuclei, **(C)** EMA +ve/−ve discrimination to choose EMA-ve nuclei **(D)** Histogram representing EMA −ve population with marker to cover nuclei and MN population, **(E)** Fluorescence vs. FSC/SSC dot plots to gate the free nuclei, **(F)** The final dot plot generated from the plots “E” to determine the nuclei, MN and hypodiploid populations (HD) respectively. **(G)** Additional plots to analyze cell cycle disruptions, and bead count can be used to determine the relative survival rate (using nuclei to bead ratio).

After incubation cells were acquired using BD Accuri™ C6 (BD Biosciences) at 488 nm excitation. EMA-associated, and SYTOX Green fluorescence were recorded in FL3 (610/20 nm) and FL1 channel (530/30 nm). In total, 10,000 gated nuclei were acquired per sample and data analysis was performed with BD Accuri™ C6 Software. Representative plots considered for MN analysis are presented in Scheme 2.

#### Evaluating Cell Cycle Perturbations

In addition to nuclei and MN detection, SYTOX Green fluorescence can be utilized to determine the cell cycle information. The gated “nuclei” population is used to analyze the cell cycle effects (see Scheme 2).

### NPs Spiking to Determine the Interferences With MN Analysis

In order to test for possible NPs interference with MN analysis, additional experiments were performed on nuclei from control cells. Thus, unexposed THP-1 cells (seeded at 1.2 × 10^5^/well) incubated at 37°C for 48 h, were centrifuged at 1,500 rpm for 5 min. Supernatant was discarded, cells were washed with chilled PBS and centrifuged to collect the pellet. Next, cells were stained with EMA followed by lysis in solution I. Along with Solution II, NiO NPs (incubated in medium at 37°C for 48 h, centrifuged at 5,000 rpm for 5 min to collect the pellet) were added and flow cytometer analysis was then performed. In addition, NiO NPs (without any nuclei) was processed for EMA staining, solution I and Solution II, similar to the protocol mentioned. These NPs were analyzed directly to determine the particle location, fluorescence in the plots, and to compare their range with MN and nuclei populations.

### Comet Assay

HBEC cells from the co-culture were assessed for DNA strand breaks by alkaline single cell comet assay as previously described (Gliga et al., 2014). Briefly, cells were mixed with 0.75% agarose (Sigma-Aldrich, St. Louis, MO) maintained at 37°C and pipetted onto precoated (0.3% agarose) microscopic slides. After gelling, the slides were transferred into lysis buffer containing 1% Triton X-100 and left overnight at 4°C. Next, slides were placed in electrophoresis buffer to unwind the DNA for 20 min, and electrophoresis was performed at 29 V for 30 min. The slides were moved into neutralizing buffer, washed in Milli-Q water, and dried overnight. Thereafter, cells were fixed in methanol for 5 min and stained with diluted (1:10,000) SYBR Green (Life Technologies™, Carlsbad, CA) in Tris-acetate-EDTA (TAE) buffer for 20 min. Slides were washed once with TAE buffer and allowed to dry before imaging. Slides were scored using a fluorescence microscope (Leica DMLB, Wetzlar, Germany) equipped with Comet Assay IV software. In total, 50 comets were counted for each sample and the DNA damage was represented as % of DNA in tail. Comets appearing as “hedgehogs” were few and were not scored. These are sometimes viewed as dead/dying cells but can also indicate damage that can be repaired (Lorenzo et al., 2013), and such comets may not be recognized by image analysis.

### Metal Release From Inserts Analyzed by ICP-MS

The metal/ion release from the inserts into lower compartment was analyzed using an inductively coupled plasma mass spectrometer, ICP-MS (ICAP Q; Thermo scientific, Waltham, MA, United States). In brief, dTHP cells (5 × 10^5^/insert) were exposed to NiO NPs at 25 μg/mL and were placed over HBEC cells for 48 h at 37°C. After incubation inserts were removed, and medium (containing dissolved Ni or possibly NiO that may be transported from the upper compartment) was collected from the HBEC wells and stored at 4°C prior to analysis. Furthermore, to analyze transport of Ni over the insert without cells, NiO NPs (25 μg/mL) were added to inserts without dTHP (acellular control) and medium was collected from HBEC cells after incubation. For analysis, samples were diluted 10 times in 2% HNO_3_ and in similar standard solutions of Ni were prepared (0, 0.1, 1, 5, 10, 50, 100, 500 ppb in 2% HNO_3_). Indium was added as an internal standard to all samples equally (5 μg/L), to enable the measured metal concentrations based on its recovery. The levels of ^58^Ni and ^115^In were quantified in each sample acquired in KED mode using argon as vector gas and helium as collision gas. The recovery of internal standard was observed between 80 and 100%. The limit of detection (LOD) was evaluated as 3 x standard deviation of blank medium samples.

## Results

### Cytotoxicity

**FIGURE 1:**
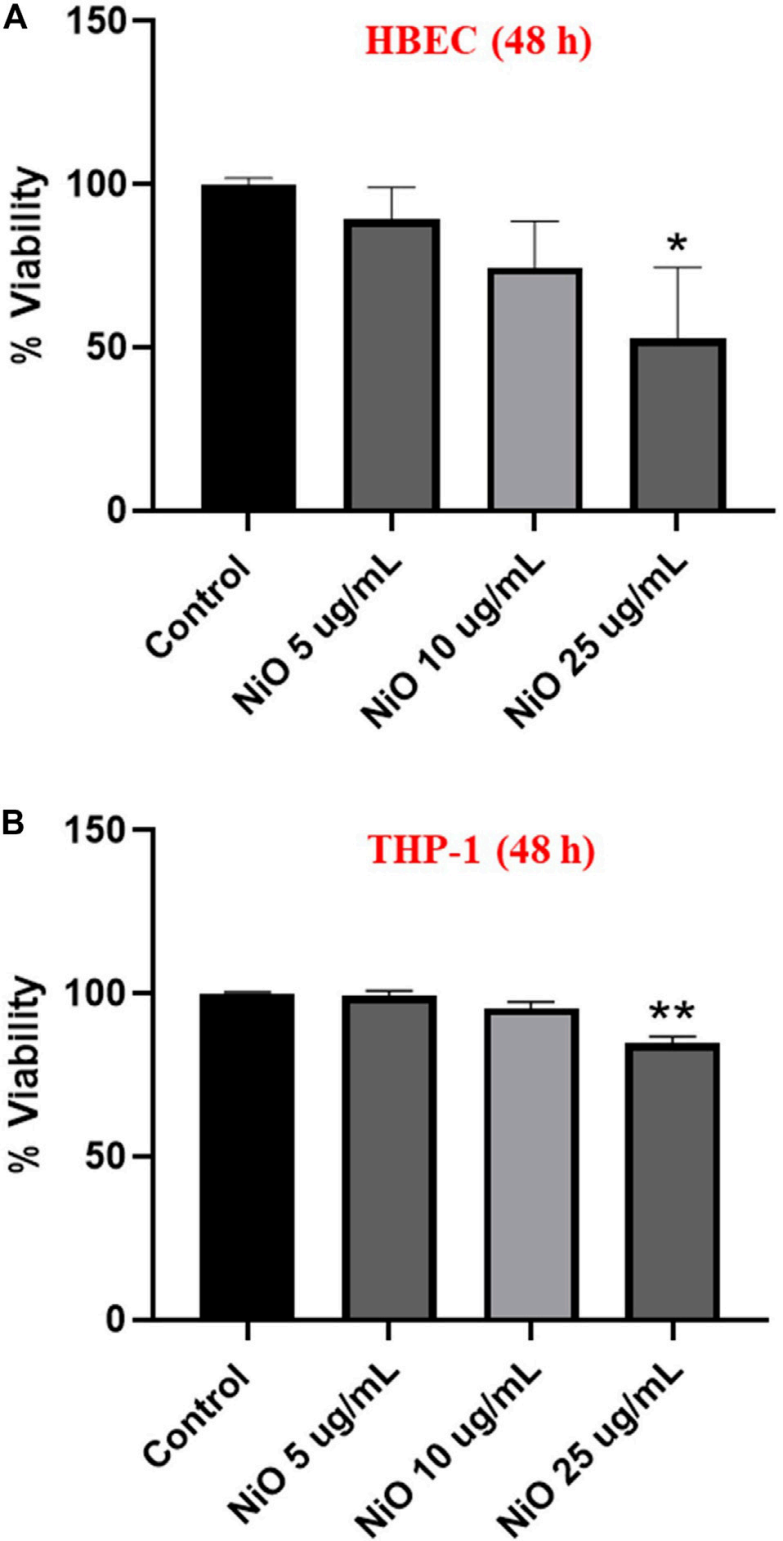
Cytotoxicity assessment of NiO NPs following exposure of HBEC cells **(A)** and THP-1 cells **(B)** after 48 h. The bars represent mean ± SEM. Asterisks indicate significance (*p < 0.05) compared to untreated cells (control).

Cell viability was assessed by using Alamar blue assay in HBEC and THP-1 monocultures after 48 h exposure to NiO NPs. In HBEC and THP-1 cells, a significant cytotoxic effect was observed at the dose 25 μg/mL NiO (Figures 1A,B).

### Primary and Secondary Genotoxicity Assessed as MN Induction Using Flow Cytometer

**FIGURE 2:**
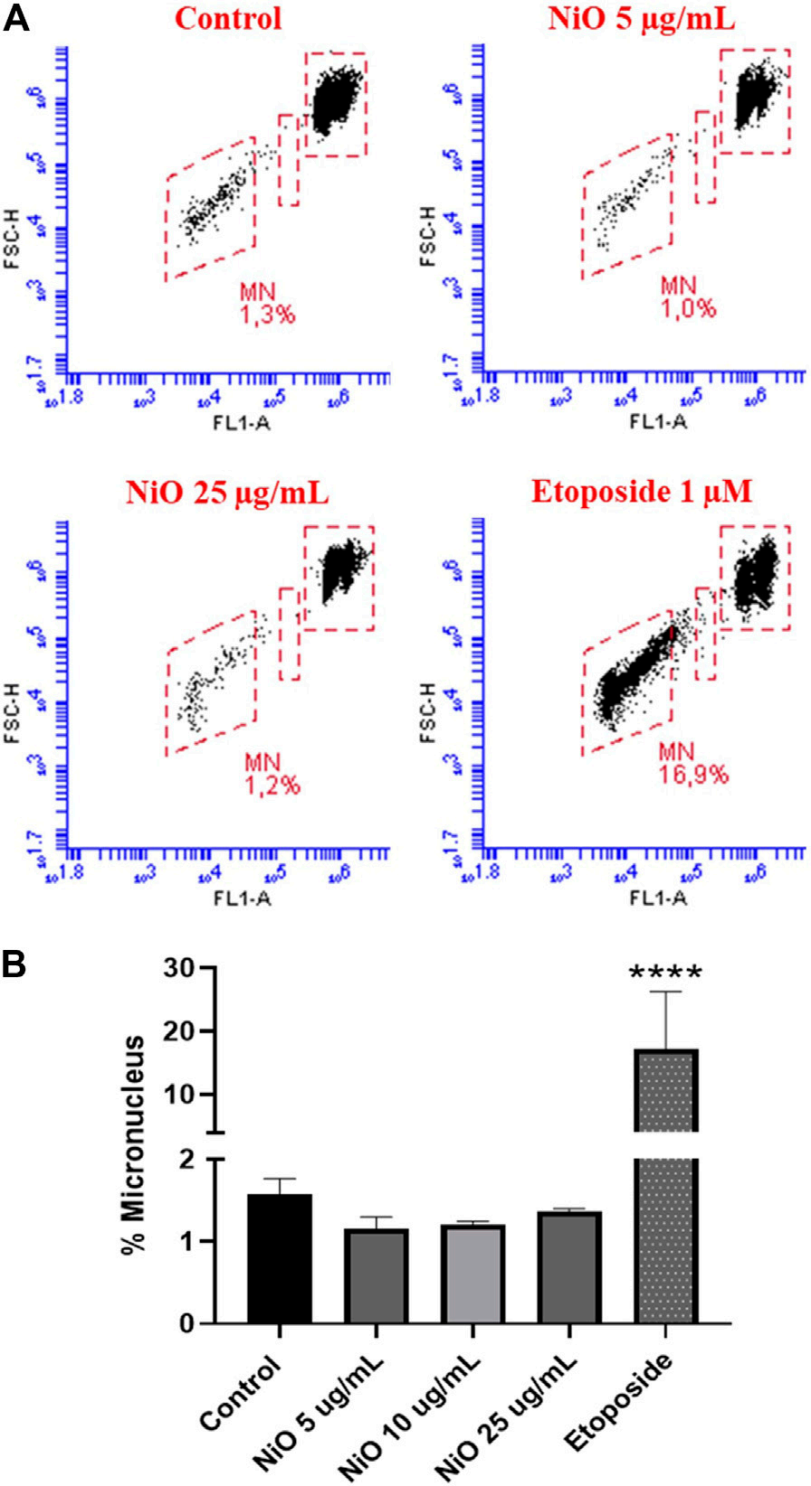
Micronucleus detection in HBEC cells exposed to NiO NPs after 48 h exposure using flow cytometric method. **(A)** Dot plot representing MN formation and **(B)** bar plot indicating MN% expressed from three individual experiments. Etoposide (1 µM) was used as a positive control. The bars represent mean ± SEM. Asterisks indicate significance (*p < 0.05) compared to untreated cells (control).

**FIGURE 3:**
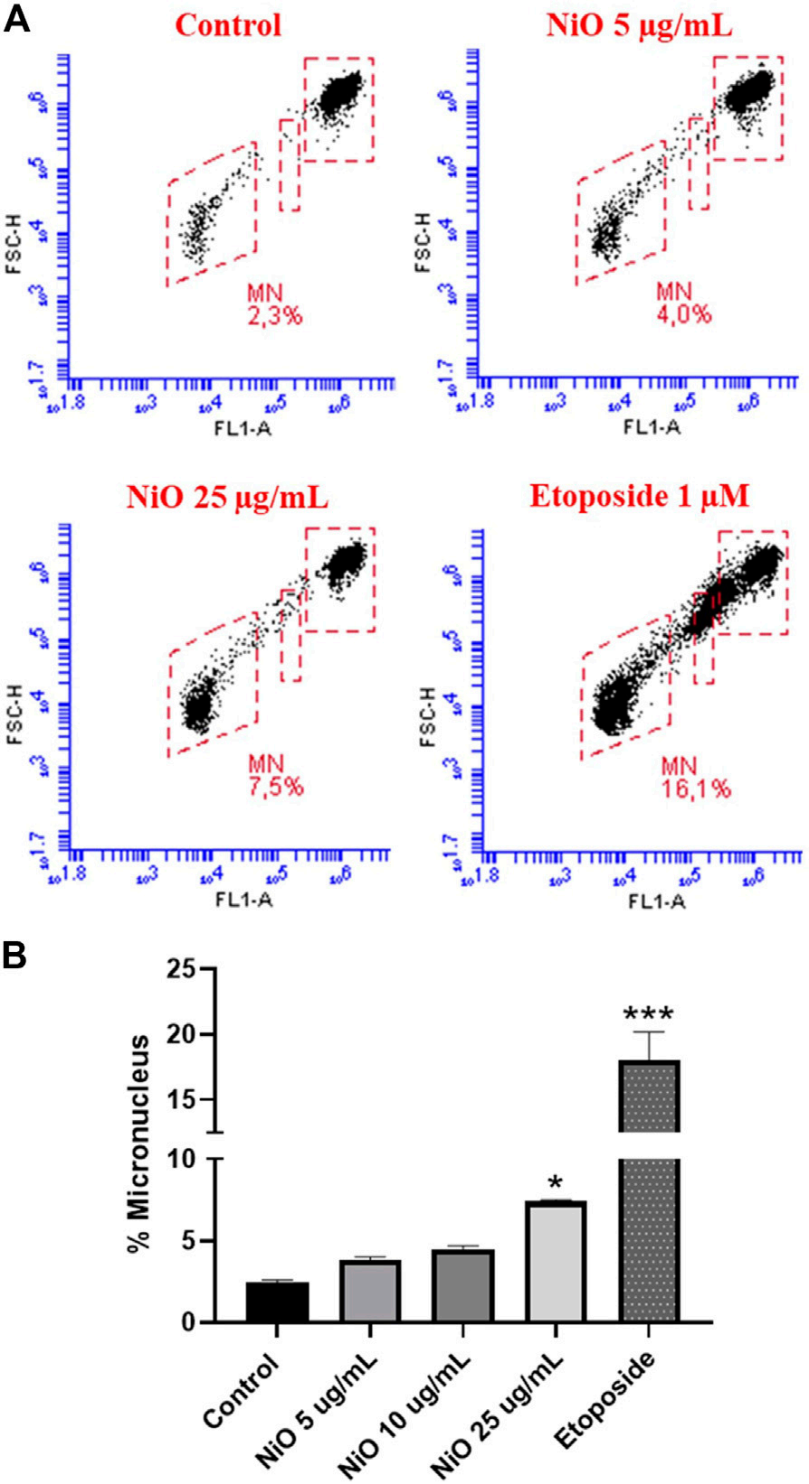
Micronucleus detection in THP-1 cells exposed to NiO NPs for 48 h using a flow cytometric method. **(A)** Dot plot representing MN formation and **(B)** bar plot indicating MN% expressed from three experiments. Etoposide (1 µM) was used as positive control. The bars represent mean ± SEM. Asterisks indicate significance (*p < 0.05) compared to untreated cells (control).

Primary genotoxicity: MN formation was evaluated after 48 h exposure of HBEC and THP-1 cells to different concentrations of NiO NPs (5, 10, and 25 μg/mL). The results showed no significant change in MN induction in HBEC cells (Figures 2A,B), whereas in THP-1 cells, a clear increase was observed (7.5% MN) at 25 μg/mL compared to control (2.3% MN) (Figure 3). The positive control etoposide (1 µM) caused a significant increase in MN formation in both HBEC (16.9% MN) and THP-1 cells (16.1% MN) after 48 h exposure (Figures 2, 3).

**FIGURE 4:**
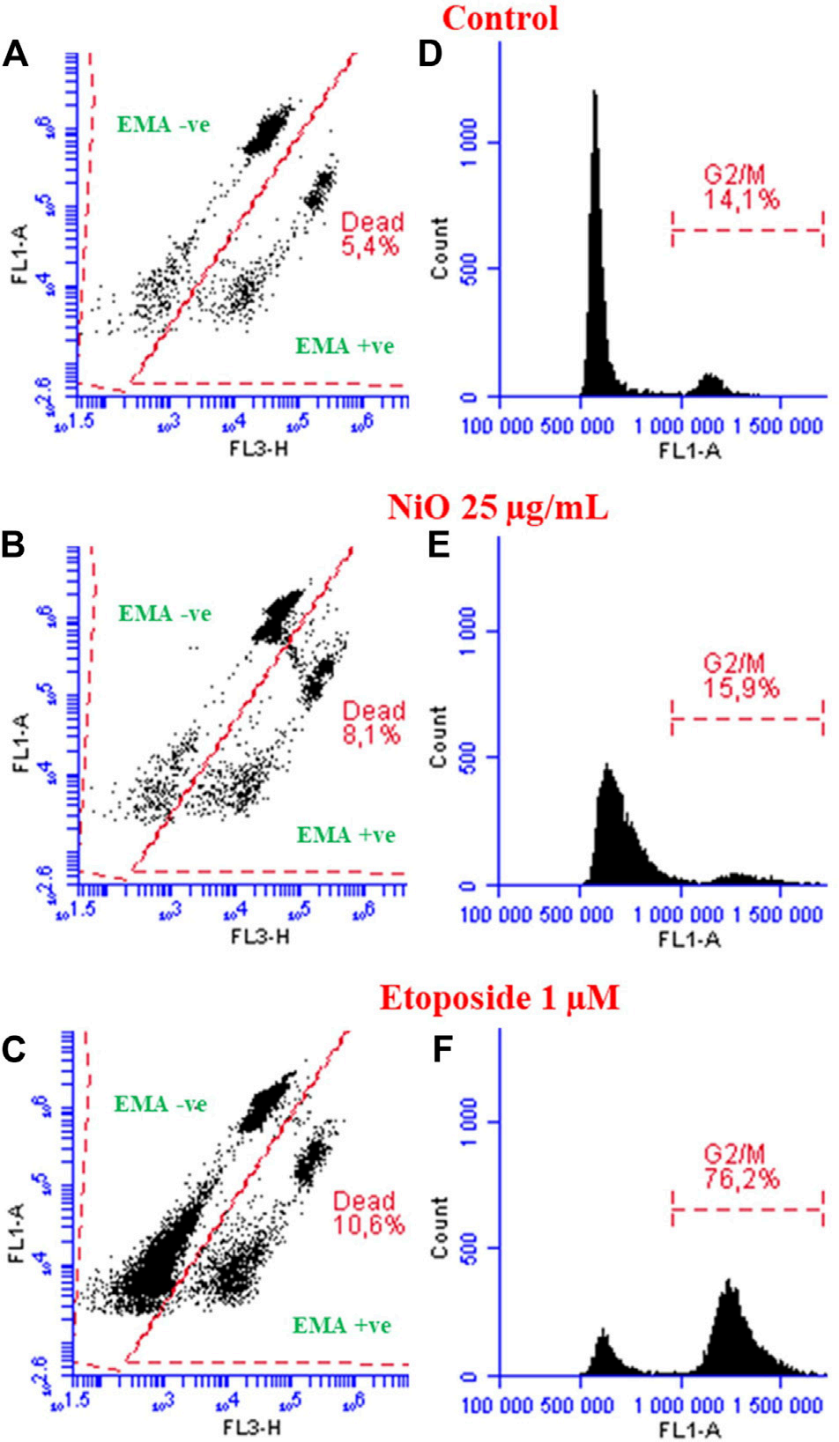
Analyzing dead population and cell cycle perturbations (HBEC cells) after exposure to NiO NPs and etoposide after 48 h. Dot plots displaying dead nuclei population (EMA +ve cells) after respective treatments compared to control **(A–C)**. Histograms represent cell cycle analysis, particularly G2/M arrest shown after NiO NPs and etoposide compared to control **(D–F)**.

The MN protocol along with micronucleus detection has an advantage to evaluate the cytotoxicity and cell cycle changes. EMA-positive nuclei, indicating nuclei from cells with comprised cell membrane, and cell cycle analysis for HBEC cells after NPs exposure is shown in Figure 4. The results showed 8.1 and 10.6% nuclei from dead cell population following exposure to NiO (25 μg/mL) and etoposide, respectively, compared to 5.4% in control cells. In addition, NiO exposure caused a slight increase in G2/M population (15.9%) and etoposide, a known cell cycle inhibitor, caused significant G2/M arrest (76.2%) compared to control 14.1% (Figure 4).

### NPs Interference Study

**FIGURE 5:**
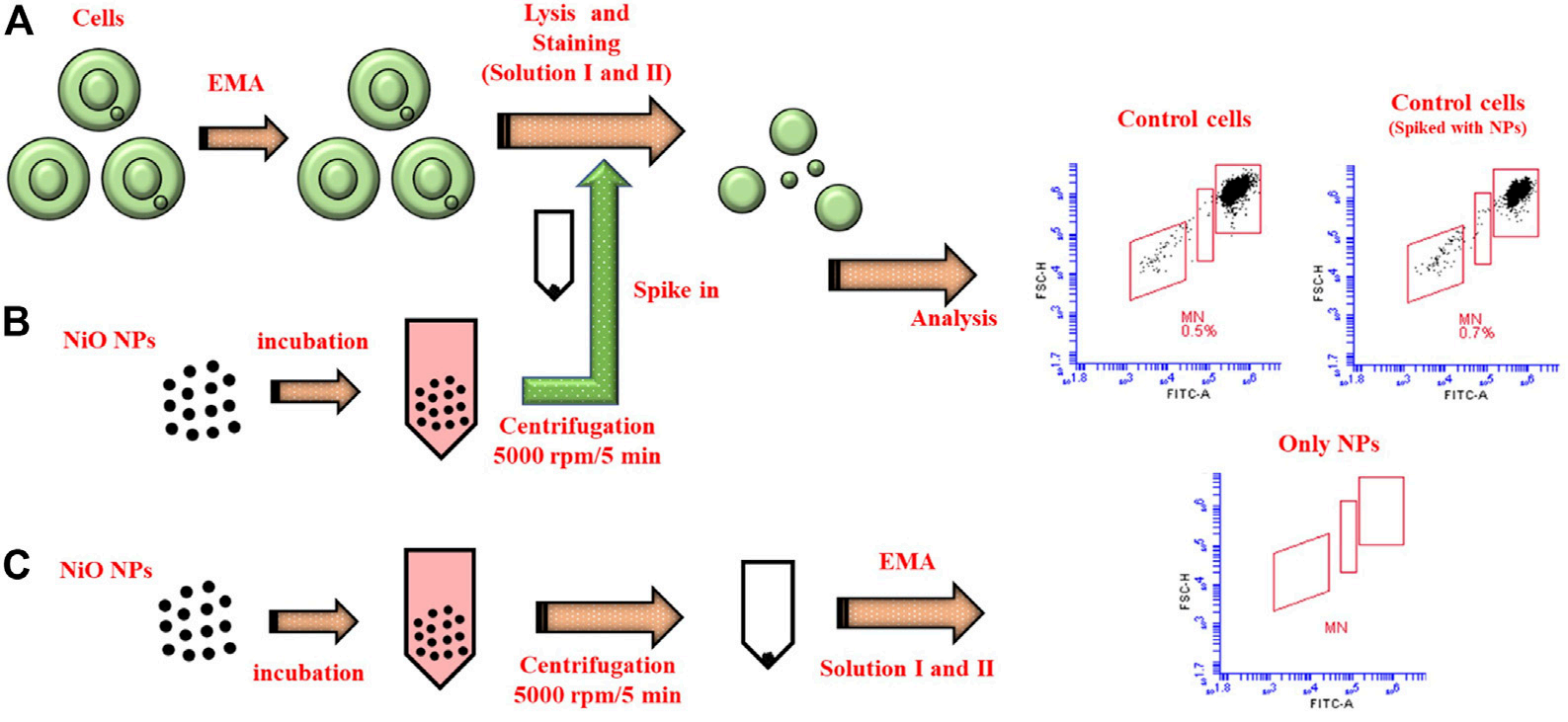
Schematic illustrating the reliability of MN protocol. **(A)** MN staining procedure carried in control cells after 48 h incubation at 37°C. **(B)** NiO NPs were incubated separately, collected through centrifugation, and spiked with the control cells to compare the interferences in MN count. **(C)** Only NiO NPs were stained following the same MN protocol and analyzed to understand their spread in the MN dot plots.

In order to elucidate possible assay interference, additional tests were performed using THP-1 cells. For this test, nuclei from control cells were compared to nuclei spiked with NPs as well as a sample containing only NPs. The results showed only a minor non-significant increase in MN in controls spiked with NPs (0.7%) compared to the control cells (0.5%). In the sample containing only NPs, there was a background observed in FSC vs. SSC dot plots (not shown), however the NPs were not detected in the gated MN and nuclei populations suggesting the reliability of the flow cytometer method (Figure 5C). Nevertheless, as different NPs possess different physico-chemical properties it is recommended to use the particle controls in parallel with samples to evaluate the interferences.

### Secondary Genotoxicity of NiO NPs (in HBEC Cells After Exposure of dTHP Cells)

**FIGURE 6:**
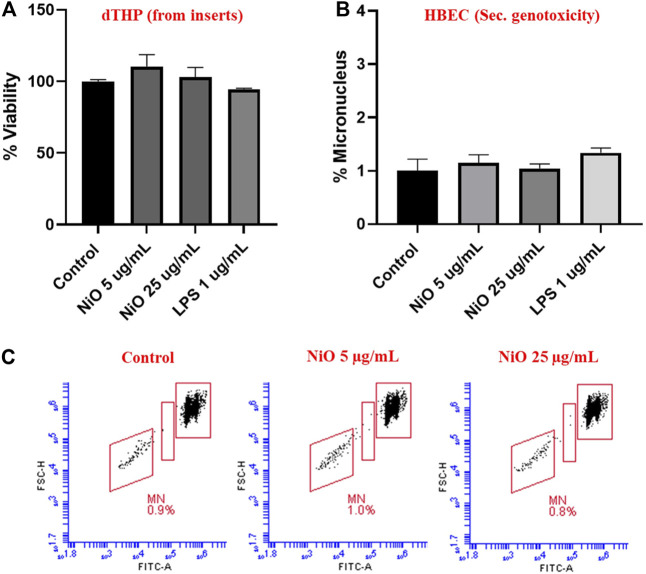
Secondary genotoxicity evaluation in HBEC cells using Flow MN method. Inserts containing dTHP were separated from co-culture and Alamar blue assay was carried to determine the cell viability after NiO NPs exposure for 48 h **(A)**. MN analysis performed in HBEC cells from the co-culture model to evaluate the secondary genotoxicity after 48 h, **(B)** bar plot representation of MN% from three experiments, and **(C)** Dot plot presentation of MN analysis from a single experiment shown in the bar plot. The bars represent mean ± SEM.

From the co-culture setup, dTHP were assessed for cytotoxicity and results indicated there was no notable change in cell viability after 48 h, which might be due to the high cell number used in the study (Figure 6A). Further, secondary genotoxicity evaluation in HBEC cells suggested no significant induction of MN in neither NiO nor LPS exposed cells compared to control cells (Figures 6B,C).

### Secondary Genotoxicity of NiO NPs in HBEC Cells Assessed by Comet Assay

**FIGURE 7:**
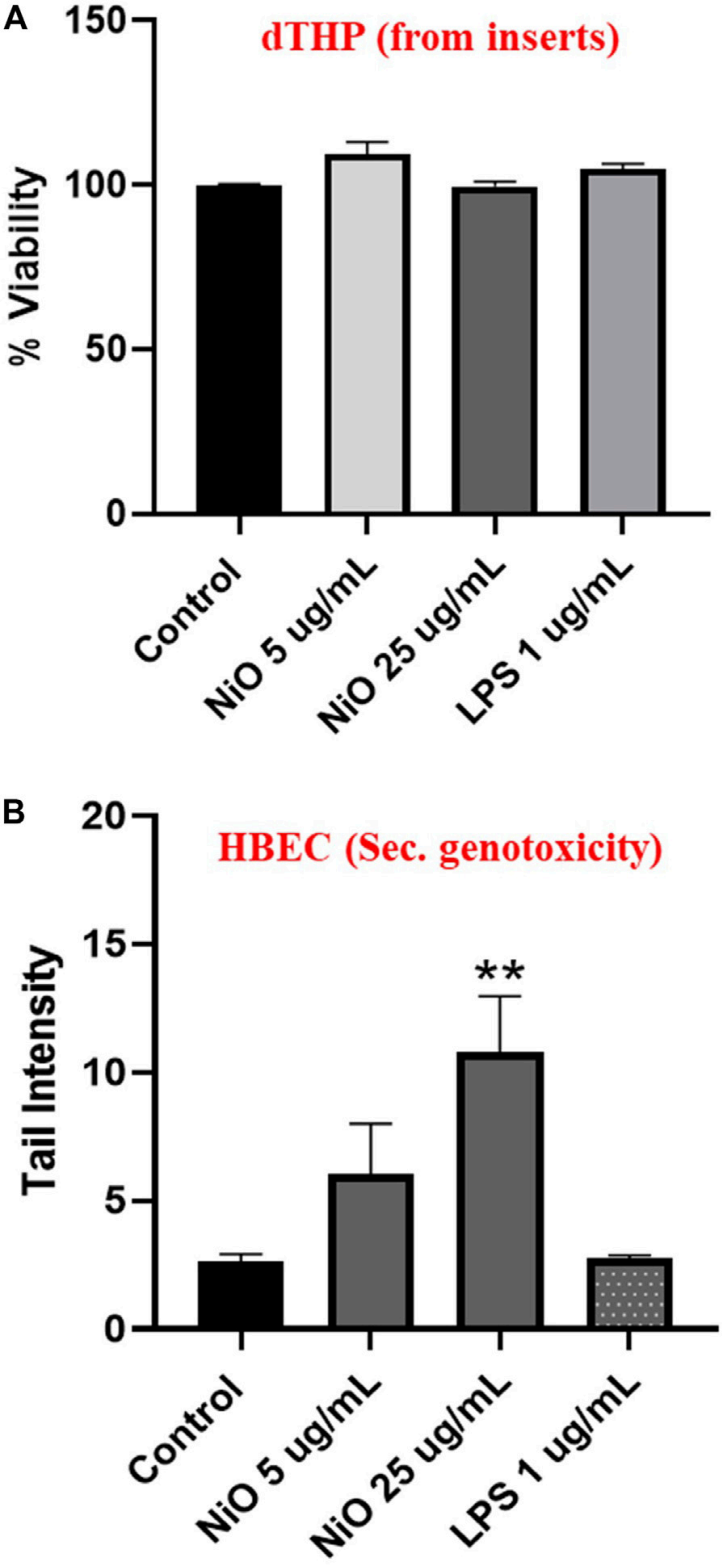
Secondary genotoxicity evaluation in HBEC cells using comet assay. Inserts containing dTHP were separated from co-culture and alamar blue assay was carried to determine the cell viability after NiO NPs exposure for 24 h **(A)**. Bar plot representing DNA damage in HBEC cells from the co-culture model to evaluate the secondary genotoxicity after 24 h **(B)**. The bars represent mean ± SEM. Asterisks indicate significance (*p < 0.05) compared to untreated cells (control).

In order to compare the MN formation with DNA strand breaks, comet assay was performed after 24 h exposure of dTHP-1 cells to NiO NPs. In line with the results from 48 h exposure, no significant cytotoxicity was observed compared to control (Figure 7A). However, secondary genotoxicity in terms of increase in DNA strand breaks in HBEC cells was observed following dTHP-1 exposure to 25 μg/mL NiO NPs. There was 2.2- and 3.8-fold increase in comet tail intensity for 5 and 25 μg/mL NiO concentrations compared to control (Figure 7B). Further, (lipopolysaccharide) LPS treatment did not show any DNA damage in HBEC cells.

### Metal Release From Inserts Analyzed by ICP-MS

In order to explore whether Ni (as NPs or ions) added to the insert in the upper compartment could be transferred to the cells in the lower compartment, ICP-MS analysis was performed to analyze Ni content of the media in the lower compartment. This was done both with and without cells (dTHP-1) on the insert. The results showed 1.1 μg/mL Ni in the media when NiO NPs was added without cells. This represents 4.4 wt% transfer of Ni to the lower compartment (data not shown). In presence of dTHP cells, the Ni content of the media 0.44 μg/mL (approx. 1.6 wt%). This suggests that some Ni was transferred (as NPs or ions) even in the presence of cells.

## Discussion

The main aim of this study was to establish a flow cytometry protocol for MN analysis that is useful for detecting primary and secondary genotoxicity of NPs. Importantly, possible interferences were also considered. NiO NPs were selected as model NPs due to our previous experience with these NPs using other methods. Our group previously showed the possibility to detect secondary genotoxicity caused by NiO NPs using “conditioned media” and co-cultures of HBEC and THP-1 (Akerlund et al., 2019). In this study, we tried further to understand the secondary genotoxicity of NiO NPs by standardizing a protocol to be used for assessing MN formation detected by flow cytometry. Moreover, this MN flow cytometric protocol was compared with comet assay to explore the secondary genotoxicity in HBEC cells co-cultured with dTHP.

Compared to single *in vitro* experiments, multi-cell models are preferable as they mimic the *in vivo* conditions better and offer an opportunity to detect a broader potential damage caused by NPs (Evans et al., 2019). Only few studies have, however, used such approaches for genotoxicity assessment of NPs or nanomaterials. For instance, a study on different iron oxide NPs evaluated the chromosomal damage by the *in vitro* micronucleus assay, and results indicated that only γ-Fe_2_O_3_ induced MN formation in lung monocultures. In contrast, immune cell conditioned media and dual cell co-culture approaches indicated that both γ-Fe_2_O_3_ and Fe_3_O_4_ NPs were genotoxic towards lung cells due to secondary genotoxicity (Evans et al., 2019). Further, genotoxic effects of few layer graphene evaluated by cytokinesis blocked micronucleus (CBMN) assay revealed significant MN induction in TT1 cells (lung cells) confirmed by both mono and co-culture approaches (Burgum et al., 2021).

To determine the genotoxicity in terms of MN formation, most of the studies use conventional microscopic methods. However, high-throughput techniques are in general getting more attention (Nelson et al., 2017) and MN detection using flow cytometry is gaining more interest. This method has the advantage to gather much information on cytotoxicity, cell cycle analysis, and MN formation in an efficient manner from the same experiments. Further, background from NPs can be minimised using cell free controls in laser-based systems, which might be difficult to interpret in microscopic analysis as NPs at higher concentrations might camouflage the MN population (Vallabani et al., 2014). Our results indicated that the THP-1 cells appeared more sensitive compared with the HBEC cells. One explanation could be a higher uptake of the particles in THP-1 cells. We did not carefully evaluate the particle internalization in this study (e.g., using TEM imaging), but in a recent study with focus on particles from 3D-printing we noted MN formation in HBEC cells by cobalt nanoparticles (used as positive control) indicating uptake of these nanoparticles (Vallabani et al., 2022).

We and others previously studied MN formation (primary genotoxicity) of various nanoparticles using flow cytometry (Di Bucchianico et al., 2017; Lebedova et al., 2018; Garcia-Rodriguez et al., 2019). Overall, these appear to be in good agreement with the microscopic method and thus, the flow cytometry version has been recommended (Garcia-Rodriges et al., 2019). Also, in previous studies we and others used *in vitro* microflow kit or similar methods; the method is easy to process and rapid in acquiring data (1.0 to 5.0 × 10^4^ nuclei per sample) compared to microscopic analysis (Bryce et al., 2008; Vallabani et al., 2014; Vallabani et al., 2019). The detailed protocol published here could be an option or complement to the kit-based method. It also offers the possibility to study cell cycle perturbations as we did for the HBEC cells, (see Scheme 2, Figure 4). Data showed minimum increase in dead population (EMA +ve) after NiO treatment (25 μg/mL) compared to control. Further, cell cycle alteration was not detected, and there was less G2/M arrest after NPs exposure. In contrast, the positive control “etoposide” a known inducer of double strand breaks triggered a significant G2/M arrest compared to control cells. Similarly, a study in A549 cells exposed to different concentrations of NiO NPs (10, 15, 50, 75, and 100 μg/mL) suggested that cell cycle alterations were only observed at higher concentrations (100 μg/mL) after 48 h (Cambre et al., 2020).

In comparison, comet assay was performed to determine the secondary genotoxicity of NiO NPs in HBEC cells after 24 h exposure. Results expressed increase in DNA damage for both 5 and 25 μg/mL treatment doses; but only the highest concentration 25 μg/mL exhibited a significant increase in tail intensity compared to control. Our earlier study showed a similar genotoxic effect in HBEC cells co-cultured with dTHP. Macrophage exposure with NiO NPs at 50 μg/mL caused a significant DNA damage in HBEC cells after 3 and 24 h (Akerlund et al., 2019). Since a minor part of the Ni (approx. 1.6 wt%) was transferred from the upper compartment with dTHP-1 cells to the lower compartment with HBEC cells, we cannot totally rule out that this affected the DNA breaks formed.

Overall, this study established a flow cytometry protocol for MN analysis that is useful for detecting primary and secondary genotoxicity of NPs. Our results also emphasize the sensitivity of THP-1 cells and thus, these may in general be a good model for assessing MN formation in future studies. Even though our analysis did not find any interference with the NPs and MN detection, it is always important to consider possible NP-assay interferences. Hence, it is also recommended to employ a set of interference controls applied for any nanomaterials and cells used in the study to improve the data reliability (Franz et al., 2020). The present study suggests that NiO NPs did not cause MN formation *via* secondary (inflammatory driven) mechanisms.

## Data Availability

The raw data supporting the conclusion of this article will be made available by the authors, without undue reservation.
